# Sequelae of Fat Grafting Postmastectomy: An Algorithm for Management of Fat Necrosis

**Published:** 2012-12-04

**Authors:** Erin L. Doren, Rajiv P. Parikh, Christine Laronga, Matthew E. Hiro, Weihong Sun, Marie Catherine Lee, Paul D. Smith, William J. Fulp

**Affiliations:** ^a^Division of Plastic Surgery, Department of Surgery, University of South Florida, Tampa; ^b^Comprehensive Breast Program, H. Lee Moffitt Cancer Center, Tampa, Florida; ^c^Department of Biostatistics, H. Lee Moffitt Cancer Center, Tampa, Florida

## Abstract

**Objective:** Fat grafting is used to improve the reconstructed breast. Local recurrences following mastectomy present as palpable subcutaneous nodules; fat necrosis/oil cysts, a sequelae of fat grafting, also present as subcutaneous nodules. Our objective was to examine the frequency and factors associated with fat necrosis in the postmastectomy reconstructed breast and propose an algorithm for management. **Methods:** A retrospective review of a breast reconstruction database was conducted to identify patients treated with fat grafting. Clinical-pathologic data at cancer presentation, volume of fat injection, occurrence of subcutaneous nodules, and outcome were collected. Statistical analysis was performed to compare the development of a palpable mass with continuous and categorical variables. **Results:** A total of 278 patients who had fat grafting with breast reconstruction were identified. Sixty-four patients (23%) developed a palpable mass at a median time of 10 months (range: 2-39). Seventeen (6%) subsequently underwent needle or excisional biopsy (16 fat necrosis/oil cysts; 1 recurrent carcinoma). The recurrent carcinoma identified did not correlate with the location of fat grafting. No clinical-pathologic (age, body mass index, stage, smoking, radiation) or technical variables (fat/tumescent volume) were found to predict development of fat necrosis. **Conclusions:** Fat grafting improves cosmesis but has a significant risk of fat necrosis. Awareness of fat injection location, incidence, time to development, and characteristic examination may decrease anxiety and need for imaging/biopsy.

In 2011, more than 96000 breast reconstructive procedures were performed in the United States.^1^ With technical advances in the type of mastectomy performed, such as the skin and nipple-sparing mastectomy and newer techniques in reconstruction, the aesthetic standards for breast reconstruction have become quite high. After primary reconstruction with implants or autologous muscle flaps, secondary procedures are often used to improve the aesthetic result. One example of this is autologous fat grafting, which is commonly used to replace subcutaneous tissue removed by mastectomy. Several studies have shown that fat transfer produces a noticeable change in size and improvement in the contour of the breast.^2^^-^4

Multiple techniques for fat grafting have been proposed and evaluated for their efficacy, but no single technique has proven superior.^5^^-^9 Techniques involve the harvesting of fat via liposuction or syringe aspiration, followed by placement of the harvested fat into varying depths of the mastectomy skin flaps. Fat is then delivered in small aliquots to increase the blood supply and chance of survival. Despite these efforts to increase graft survival, graft loss and subsequent fat necrosis occur in 2% to 18% of fat grafting procedures.^3^^,^^4^^,^^9^^-^14

Local recurrences following mastectomy present as palpable nodules in the skin or subcutaneous tissue.^15^ Fat necrosis/oil cysts may also present as a subcutaneous nodule in the surgical field. It is common practice for breast surgeons to counsel patients to perform self–breast examinations after mastectomy and to be concerned about skin changes or palpable nodules under the native breast skin. Not uncommonly in our practice, women return to their breast surgeon, with the complaint of a new palpable nodule or small cyst in the area of their previous fat grafting. These patients are most often referred for a breast ultrasound or needle biopsy; any abnormal or suspicious findings are then further evaluated or surgically excised. Many of these patients proceed to core needle or excisional biopsies to definitively rule out the presence of cancer. In our experience, palpable nodules or cysts in the fat grafted area have always been found to be benign. The procedures involved to rule out cancer recurrence can cause patients undue psychologic stress and subject them to additional operative intervention.

The current literature on the outcomes of fat grafting for breast reconstruction is sparse; we look to better characterize the sequelae of fat grafting and the incidence of fat necrosis/oil cysts to hone clinical management of patients treated with this relatively new technique. Our specific goals are as follows: (1) delineate an algorithmic approach to manage fat grafting complications, (2) improve patient selection, and (3) evaluate the oncologic safety of fat grafting after breast cancer treatment.

## METHODS

### Demographics

This is an institutional review board–approved, retrospective review of a prospective breast reconstruction database. At Moffitt Cancer Center, from 2005 to 2010, 1289 patients had breast reconstruction. A search was conducted to identify patients having autologous fat grafting procedures and identified 286 patients who had fat grafting by a single reconstructive surgeon (P.D.S.). Patients having lumpectomy and fat grafting were excluded from this study. Patient factors hypothesized to impact development of fat necrosis, such as body mass index, history of smoking, comorbid diabetes and/or hypertension, and family or personal histories of breast cancer were recorded. In addition, cancer characteristics, including stage, tumor type, and receipt of postmastectomy radiation, were documented. Patient demographic and clinical data are presented in Table 1.

### Surgical technique

All fat grafting procedures were performed or supervised by a single plastic surgeon (P.D.S.). Areas of contour deformity in the reconstructed breast were marked preoperatively. Fat was most commonly harvested from the lateral thigh, abdomen, or flank. Prior to lipoaspiration, the donor sites were infiltrated with tumescent solution (1000 mL of saline, 10 mL of 1% lidocaine, and 1:100 000 epinephrine). Fat was aspirated by conventional liposuction technique utilizing a blunt cannula attached to a liposuction machine. Harvested fat was immediately transferred into 10-mL syringes, capped and allowed to purify by gravity sedimentation. After discarding oil and debris, small aliquots of fat were injected subdermally through small incisions in the premarked areas of the reconstructed breast. The location and amount of fat injected were recorded in the patient's operative report.

### Postoperative surveillance and follow-up

Patients return to Moffitt Cancer Center after breast reconstruction at regular intervals to be evaluated by both the breast and plastic surgery teams. Any small palpable nodule or cyst found on physical examination was appropriately documented. If nodules were subcutaneous, small (<5 mm), not associated with any skin changes or lymphadenopathy, and found in the area of fat grafting, a decision was made to follow the lesion clinically or to have an ultrasound. Patients with any suspicious lesion—change in skin color, induration, larger nodules, lymphadenopathy, or lesion in location other than fat grafting—were immediately referred to targeted ultrasound by a fellowship trained breast radiologist and further workup was performed as deemed necessary. For this study, the number, size, orientation, location, and breast imaging-reporting and data system (BI-RADS) classification of nodules were recorded. Histopathology was recorded for all patients undergoing core or excisional biopsy. Any local, regional, or distant recurrences of disease were similarly documented.

### Statistical analysis

Along with descriptive statistics, we evaluated the association between various clinical and breast reconstruction variables and the development of a palpable mass. The unit of this analysis was at the patient level, with development of a palpable mass being considered true if either breast developed a palpable mass. Because many patients had bilateral mastectomy with reconstruction, and multiple procedures, fat graft data were evaluated and presented per breast mound. Breast reconstruction-specific variables, such as amount of fat and tumescent injected, were averaged across the reconstructions for each patient. Wilcoxon rank sum test and exact chi-square test using Monte-Carlo estimation approach were used to compare the development of a palpable mass with continuous and categorical variables, respectively. All *P* values are 2-sided tests and declared as significant at a .05 level. SAS 9.3 (SAS Institute Inc, Cary, NC) was used for the analysis.

## RESULTS

Autologous fat grafting for postmastectomy breast reconstruction was performed in 278 patients. Because of bilateral and multiple procedures, there were a total of 448 reconstructed breasts and 586 fat grafting procedures. Definitive breast cancer surgery, adjuvant chemotherapy, and radiation therapy were accounted for as treatment interventions and summarized in Table 2. Of the patients undergoing bilateral mastectomy (190 patients and 380 breasts), 203 (53%) mastectomies were prophylactic; 16 (8%) patients had mastectomy for bilateral breast cancer, 140 (74%) had unilateral breast cancer with contralateral prophylactic mastectomy, and 32 (17%) had bilateral prophylactic mastectomies (2 patients with data not documented). Oncologic procedures are presented in Table 3. Of the 244 women who had mastectomy for cancer treatment (not prophylactic), 36 (15%) were stage 0, 93 (38%) stage I, 73 (30%) stage II, 19 (8%) stage III, and 2 (0.8%) stage IV (21 patients with data not documented).

**Figure 1:**
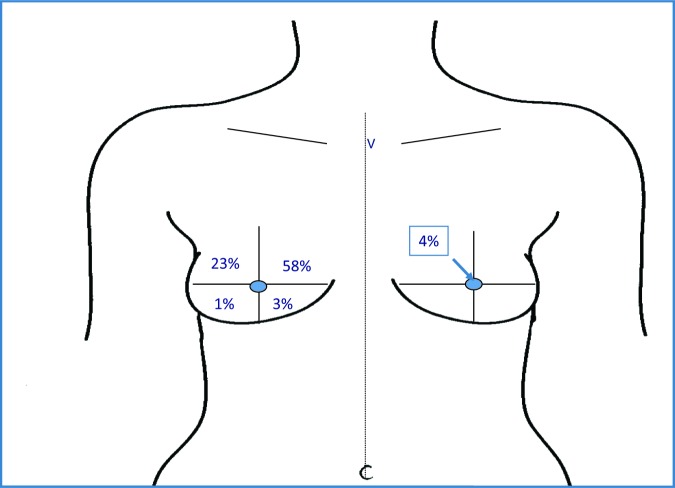
Area of contour abnormality requiring fat grafting.

Primary mode of breast reconstruction is shown in Table 4. The most common reconstructive procedure was tissue expander with permanent implant reconstruction (63%), followed by latissimus dorsi muscle flap with tissue expanders (16%). Reconstruction was immediate in 80% (357 of 448) of cases and delayed in 20% (91 of 448). Of the patients having autologous fat grafting, 72% had fat grafting performed once, 25% had 2 procedures, and 3% had 3 or more procedures (average 2.04 procedures). For the majority of patients, performance of fat grafting was conducted at the time of tissue expander to permanent implant exchange, or concurring with nipple areolar reconstruction (32% and 35%, respectively). The average time from mastectomy to fat grafting was 16.7 months. The average volume of fat injected was 50 mL (range: 5-200 mL). The most common location for fat grafting was the upper inner quadrant of the breast (58%) (Fig 1).

A total of 64 patients (23%) developed a palpable mass at a median time of 10 months (range: 2-39) from fat grafting. Twenty-nine patients (10%) had a diagnosis of fat necrosis rendered on clinical examination alone, whereas 35 patients (13%) had ultrasound performed in addition to examination. Ultrasound imaging revealed 31% with BI-RADS 2, 20% BI-RADS 3, and 49% BI-RADS 4. Seventeen patients (6%) went on to tissue diagnosis; 14 patients via needle biopsy, 2 via excisional biopsy, and 1 patient via core biopsy of 1 breast and excisional biopsy of the other breast. The location of the palpable nodules correlated with the area fat grafted in 47 patients, and the remaining 17 patients all required tissue diagnosis. The results confirmed oil cyst or fat necrosis in all but 1 patient. This patient had a recurrent carcinoma remote from the location of fat grafting. Ultrasound imaging of this patient revealed classic fat necrosis in the upper inner quadrant (area of known fat grafting, biopsy proven fat necrosis) but demonstrated suspicious findings in the second palpable area (lower outer quadrant, outside area of fat grafting, and biopsy proven recurrent cancer).

Median follow-up was 28 months (range: 0.56-168 months). At most recent follow-up, 3 patients developed distant metastatic disease, 6 had a documented locoregional cancer recurrence (2.2%), and 1 died of disease. No clinical-pathologic (age, body mass index, stage, radiation, tobacco use) or technical variables (fat/tumescent volume) were found to predict development of fat necrosis (Table 5).

The efficacy of fat grafting was reviewed at the time of last follow-up. At their most recent visit, most patients were asked to rate their satisfaction with final reconstruction. Patient-reported satisfaction with breast reconstruction was as follows: 31% very satisfied, 36% mostly satisfied, 6% satisfied, and 2% dissatisfied. At last follow-up, the plastic surgeon reports breast reconstruction cosmesis to be 25% excellent, 44% good, 4% fair, and 1% poor.

## DISCUSSION

With improvements in surgical technique and materials, patient and surgeon desire for improved aesthetic results are reflected in secondary breast reconstruction procedures and refinements.^16^^,^^17^ Of particular significance to patient satisfaction is the reconstruction of natural contour, shape, and symmetry at the culmination of the breast reconstructive process.^18^ Despite advances in reconstructive technique, secondary contour deformities can still occur and result in an aesthetic outcome short of patient expectations. Autologous fat grafting for soft-tissue augmentation has gained increasing popularity as a technique to improve shape and resolve contour deformities of the reconstructed breast and chest wall.^2^^-^4 The indications for fat transfer to the breast are constantly expanding and even include reconstruction with fat transplantation alone.^19^ However, despite increasing popularity, several concerns persist about fat grafting. In evaluating any intervention, the safety, as manifested by the incidence and nature of complications, is a primary concern. To our knowledge, this study is one of the largest with long-term follow-up data to evaluate fat grafting and its sequelae.

### Complications

As with any surgical procedure, autologous fat grafting has associated complications. While major complications are exceedingly rare, minor complications of fat necrosis/oil cysts are consistently reported. Fat necrosis is a complication of several breast procedures, including biopsy, reduction, augmentation, and reconstruction.^20^^-^23 The most accepted theory is that insufficient vascularity and ischemia are primarily responsible for the development of fat necrosis. Other complications associated with fat grafting are low and include seroma, infection, and liposuction-related pneumothorax, none of which were found in our study.^12^^,^^13^

Rates of graft-related fat necrosis have been reported between 2% and 18%.^3^^,^^4^^,^^9^^-^14 In our series, the incidence of fat necrosis was notably higher at 23%. This may be attributed to our increased postoperative surveillance and criteria for fat necrosis reporting. In our institution, patients are monitored at routine intervals by their breast and plastic surgeon providers (every 6 months for 5 years, and every 6 months for first year and then yearly, respectively). Once palpable nodules or cysts were compared to the locations fat grafted, the fat necrosis rate directly related to grafting was 16.9%, as not all nodules were found at sites grafted. Other palpable nodules are attributed to necrosis secondary to other breast procedures including mastectomy, autologous muscle flap, or expander placement; all of these patients required ultrasound or biopsy to rule out cancer.

**Figure 2:**
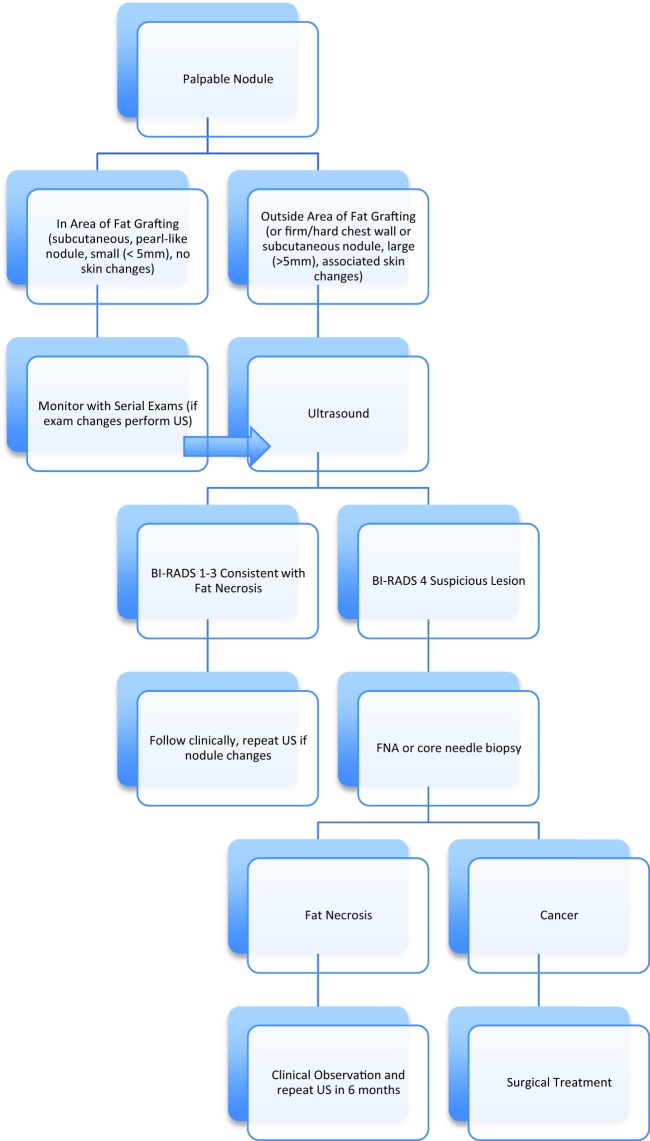
Algorithm for management of a palpable nodule. BI-RADS indicates breast imaging-reporting and data system; FNA, fine needle aspiration; US, ultrasound.

Understanding that fat necrosis and oil cysts can occur after fat grafting procedures, the next step was to analyze whether we could differentiate these minor complications from recurrent cancer. Of specific concern was our ability to differentiate palpable nodules as sequelae of fat grafting from palpable nodules related to recurrent disease. There are currently no National Comprehensive Cancer Network guidelines that define a screening protocol for the reconstructed breast. Physical examination is the most accepted method to reliably screen for recurrent cancer.^24^^,^^25^ Clinical assessment has also been suggested to be an accurate method for detecting fat necrosis presenting as palpable lesions.^26^ However, a strict protocol must be followed if clinical evaluation is the primary determinant of fat necrosis. Patients must return for follow-up examinations at regular intervals, and examinations must be performed by an experienced breast surgeon. The presence, size, and location of fat necrosis should be consistently documented. New nodules, nodules occurring in areas that were not injected with fat, nodules increasing in size, or suspicious nodules detected on clinical examination should undergo further workup. We, therefore, propose the algorithm in Figure 2 as a reference to guide physicians in the care of a patient with a palpable nodule after fat grafting for reconstruction.

### Clinical-patholgic and technical variables

In our study, clinical-pathologic and technical data were assessed to identify any risk factors that may complicate results of fat grafting, aiding in our future patient selection. Diabetes, hypertension, and smoking are established risk factors for poor microcirculation and therefore, in theory, should contribute to an increased incidence of fat necrosis. However, our results challenge the veracity of this assumption in the clinical population. We found that diabetes, hypertension, and smoking were not significant risk factors for complications (Table 5). This is consistent with prior studies that show that medical comorbidities make no contribution to complication rates.^9^^,^^10^ Our results show that patients with these comorbidities can benefit from fat grafting procedures and should not be excluded when selecting patients.

The impact radiation therapy has on the outcome of fat grafting is controversial. Some purport that a previously irradiated breast creates a “poor recipient bed” for fat grafting.^2^ Rietjens et al^11^ report a higher incidence of complications with previously irradiated patients. However, clinical studies have shown no significant association between a history of radiation therapy and complications. We observed no correlation between a history of breast radiation and complications (Table 5). Experimental studies investigating the effects of radiation on fat grafts histology are warranted to lend clarity to these findings.

It has been suggested, though not clinically proven for the breast in vivo, that injecting large quantities of fat to a deficit or “overcorrection” may contribute to fat necrosis because the graft exceeds the recipient vascular supply. To counteract this, we utilize the well-established principle of injecting small aliquots of fat in multiple passes.^9^ Of note, we found that neither the volume of tumescent solution used nor the amount of fat injected to a particular site was significantly associated with an increased incidence of fat necrosis (Table 5).

### Oncologic safety

In patients with a history of breast cancer, oncologic safety is paramount. Concerns have been propagated from in vitro studies suggesting a potentially deleterious interplay between grafted fat and the recipient tissue. In 2009, the American Society of Plastic Surgeons reported findings from a task force investigating the safety and efficacy of fat grafting. Although unable to make specific recommendations for the use of fat grafting, the task force concluded that there is a risk of the procedure interfering with breast cancer detection.^9^ The task force recommended concentrating future research efforts on studies that identify risk factors for fat grafting procedures and studies that assess the effect of fat grafting on breast cancer detection and treatment.^9^ Lohsiriwat et al^27^ recently summarized a plethora of experimental studies and found that adipocyte, adipokines, and other adipocyte secretions can potentially promote breast cancer tumorgenesis by enhancing tumor-stroma interactions. Despite this, no in vivo studies demonstrate the effect of fat grafting on breast cancer development, progression, or local-regional recurrence. In our experience, only 1 patient was found to have recurrent breast cancer after fat grafting, and the recurrence was remote from the fat graft site. All other nodules occurring in areas of fat grafting were ultimately found to be benign, either by biopsy or by demonstrating clinical and radiographic stability over time. The incidence of local-regional recurrence in breast cancer treatment/reconstructive patients is reported to be between 2.2% and 8.1%.^12^^,^^13^^,^^15^^,^^25^^,^^28^^,^^29^ The local-regional recurrence rate in our study was low at 2.2%. Therefore, we found no association between fat grafting and increased occurrence or recurrence of cancer. Of note, however, is that our study is limited by a median follow-up of 28 months.

## CONCLUSION

Fat grafting improves cosmesis after postmastectomy breast reconstruction but has a significant risk (16.9%) of fat necrosis/oil cyst development. Patients should be adequately informed of the sequelae of fat grafting and the possible need for future ultrasounds and biopsy should fat necrosis occur. Awareness of fat injection location, incidence, expected time to development, and characteristic examination may decrease patient anxiety and need for imaging/biopsy. To this end, we have developed an algorithm to guide the practitioner in the management of a palpable nodule in this cohort of patients. We believe that continued research into analyzing the radiologic features of palpable nodules with ultrasound and development of standards for radiologic evaluation with characteristic descriptors to safely classify fat necrosis is warranted. New imaging standards, beyond the existing BI-RADS classification, may be necessary to accurately separate fat necrosis from malignancy in patients who have had fat grafting.

## Figures and Tables

**Table 1:** Demographic and clinical data

Patient population	278
Mean age (range), y	51 (21-81)
Race, *n* (%)	
Caucasian	241 (87%)
African American	16 (6%)
Hispanic	19 (7%)
Asian	1 (0.4%)
Mean body mass index (range)	26.69 (19-48.7)
Sex, *n* (%)	
Female	277 (99.6%)
Male	1 (0.4%)
Family history of breast cancer	141 (51%)
Personal history of previously treated breast cancer	97 (35%)
Tobacco use (within the last year)	33 (12%)
Hypertension	60 (22%)
Diabetes	13 (5%)

**Table 2:** Treatment interventions

	No. of patients
Unilateral mastectomy	88 (32%)
Bilateral mastectomy	190 (68%)
Postmastectomy radiation treatment	34 (12%)
Premastectomy radiation	56 (20%)
Adjuvant chemotherapy	78 (28%)

**Table 3:** Oncologic surgical procedure (per breast that was fat grafted)

	No./448 breasts
Skin sparing mastectomy (SSM)	90 (20%)
SSM with sentinel lymph node biopsy (SLNB)	216 (48%)
Nipple sparing mastectomy (NSM)	13 (3%)
NSM with SLNB	19 (4%)
Total mastectomy (TM)	12 (3%)
TM with SLNB	38 (8%)
Modified radical mastectomy	31 (7%)
Data not documented	26 (6%)
Other	3 (0.7%)

**Table 4:** Type of reconstructive procedure (per breast that was fat grafted)

	No./448 breasts
Tissue expander (TE) reconstruction	280 (63%)
Pedicled transverse rectus abdominus muscle flap (TRAM)	63 (14%)
Free TRAM	6 (1.3%)
Latissimus dorsi flap with TE	72 (16%)
Latissimus dorsi flap with implant sparing	2 (0.4%)
Deep inferior epigastric artery flap	10 (2%)
Immediate implant placement	13 (2.9%)
Implant sparing	1 (0.2%)
Fat grafting alone	1 (0.2%)

**Table 5:** Independent variables versus fat necrosis

	P*
Body mass index	.2181
Age	.2961
Average amount fat grafted	.3302
Average amount of tumescent injected	.1075
History of diabetes	.5042
History of hypertension	.2268
Current or recent use of tobacco	.2867
Prior breast radiation	.8605
Cancer stage	.1879

* *P* value is calculated using Wilcoxon rank sum test using exact method with Monte-Carlo estimation.

## References

[1] American Society of Plastic Surgeons Report of the 2011 Plastic Surgery Statistics. http://www.plasticsurgery.org..

[2] Kanchwala SK, Glatt BS, Conant EF, Bucky LP (2009). Autologous fat grafting to the reconstructed breast: the management of acquired contour deformities. Plast Reconstr Surg.

[3] Coleman SR, Saboeiro AP (2007). Fat grafting to the breast revisited: safety and efficacy. Plast Reconstr Surg.

[4] Spear SL, Wilson HB, Lockwood MD (2005). Fat injection to correct contour deformities in the reconstructed breast. Plast Reconstr Surg.

[5] Smith P, Adams WP Jr, Lipschitz AH (2006). Autologous human fat grafting: effect of harvesting and preparation techniques on adipocyte graft survival. Plast Reconstr Surg.

[6] Bucky LP, Kanchwala SK (2007). The role of autologous fat and alternative filler in the aging face. Plast Reconstruc Surg.

[7] Pu LLQ, Coleman SR, Cui X (2008). Autologous fat grafts harvested and refined by the Coleman technique: a comparative study. Plast Reconstr Surg.

[8] Crawford JL, Hubbard BA, Colbert SH, Puckett CL (2010). Fine tuning lipoaspirate viability for fat grafting. Plast Reconstr Surg.

[9] Gutowski KA, Baker SB, Coleman SR (2009). Current applications and safety of autologous fat grafts: a report of the ASPS Fat Graft Task Force. Plast Reconstr Surg.

[10] Losken A, Pinell XA, Sikoro K (2011). Autologous fat grafting in secondary breast reconstruction. Ann Plast Surg.

[11] Rietjens M, De Lorenzi F, Rossetto F (2011). Safety of fat grafting in secondary breast reconstruction after cancer. J Plast Reconstr Aesthet Surg.

[12] Petit JY, Lohsiriwat V, Clough KB (2011). The oncological outcome and immediate surgical complication of lipofilling in breast cancer patients: a multicenter study, Milan-Paris-Lyon experiences of 646 lipofilling procedures. Plast Reconstr Surg.

[13] Delay E, Garson S, Tousson G, Sinna R (2009). Fat injection to the breast: technique, results, and indications based on 880 procedures over 10 years. Aesthet Surg J.

[14] Missana MC, Laurent I, Barreau L, Balleyguier C (2007). Autologous fat transfer in reconstructive breast surgery: indications, technique and results. Eur J Surg Oncol.

[15] Langstein HN, Cheng MH, Singletary SE (2003). Breast cancer recurrence after immediate reconstruction: patterns and significance. Plast Reconstr Surg.

[16] Beahm EK, Walton RL (2007). Revision in autologous breast reconstruction: principles and approach. Clin Plast Surg.

[17] Enajat M, Smit JM, Rozen WM (2010). Aesthetic refinements and reoperative procedures following 370 consecutive DIEP and SIEA flap breast reconstructions: important considerations for patient consent. Aesthet Plast Surg.

[18] Snell L, McCarthy C, Klassen A (2010). Clarifying the expectations of patients undergoing implant breast reconstruction: a qualitative study. Plast Reconstr Surg.

[19] Del Vecchio DA, Bucky LP (2011). Breast augmentation using preexpansion and autologous fat transplantation: a clinical radiographic study. Plast Reconstr Surg.

[20] Chala LF, de Barros N, de Camargo Maraes P (2004). Fat necrosis of the breast: mammographic, sonographic, computed tomography, and magnetic resonance imaging findings. Curr Probl Diagn Radiol.

[21] Mandrekas AD, Assimakopoulos GI, Mastorakos DP, Pantzalis K (1994). Fat necrosis following breast reduction. Br J Plast Surg.

[22] Handel N, Jensen JA, Black Q (1995). The fate of breast implants: a critical analysis of complications and outcomes. Plast Reconstr Surg.

[23] Kroll SS (2000). Fat necrosis in free transverse rectus abdominis myocutaneous and deep inferior epigastric perforator flaps. Plast Reconstr Surg.

[24] Zakhireh J, Fowble B, Esserman LJ (2010). Application of screening principles to the reconstructed breast. J Clin Oncol.

[25] Reddy S, Colakoglu S, Curtis MS (2011). Breast cancer recurrence following postmastectomy reconstruction compared to mastectomy with no reconstruction. Ann Plast Surg.

[26] Hassa A, Curtis MS, Colakoglu S, Tobias AM, Lee BT (2010). Early results using ultrasound-assisted liposuction as a treatment for fat necrosis in breast reconstruction. Plast Reconstr Surg.

[27] Lohsiriwat V, Curigliano G, Rietjens M (2011). Autologous fat transplantation in patients with breast cancer: “silencing” or “fueling” cancer recurrence?. The Breast.

[28] McCarthy CM, Pusic AL, Sclafani L (2008). Breast cancer recurrence following prosthetic, postmastectomy reconstruction: incidence, detection, and treatment. Plast Reconstr Surg.

[29] Howard MA, Polo K, Pusic AL (2006). Breast cancer local recurrence after mastectomy and TRAM flap reconstruction: incidence and treatment options. Plast Reconstr Surg.

